# Albatrosses Following Fishing Vessels: How Badly Hooked Are They on an Easy Meal?

**DOI:** 10.1371/journal.pone.0017467

**Published:** 2011-03-02

**Authors:** José P. Granadeiro, Richard A. Phillips, Paul Brickle, Paulo Catry

**Affiliations:** 1 CESAM, Museu Nacional de História Natural, Universidade de Lisboa, Lisboa, Portugal; 2 British Antarctic Survey, Natural Environment Research Council, Cambridge, United Kingdom; 3 Falkland Islands Government, Directorate of Natural Resources, Fisheries Department, Stanley, Falkland Islands; 4 Eco-Ethology Research Unit, ISPA, Lisboa, Portugal; Institute of Marine Research, Norway

## Abstract

Fisheries have major impacts on seabirds, both by changing food availability and by causing direct mortality of birds during trawling and longline setting. However, little is known about the nature and the spatial-temporal extent of the interactions between individual birds and vessels. By studying a system in which we had fine-scale data on bird movements and activity, and near real-time information on vessel distribution, we provide new insights on the association of a threatened albatross with fisheries. During early chick-rearing, black-browed albatrosses *Thalassarche melanophris* from two different colonies (separated by only 75 km) showed significant differences in the degree of association with fisheries, despite being nearly equidistant to the Falklands fishing fleet. Most foraging trips from either colony did not bring tracked individuals close to vessels, and proportionally little time and foraging effort was spent near ships. Nevertheless, a few individuals repeatedly visited fishing vessels, which may indicate they specialise on fisheries-linked food sources and so are potentially more vulnerable to bycatch. The evidence suggests that this population has little reliance on fisheries discards at a critical stage of its nesting cycle, and hence measures to limit fisheries waste on the Patagonian shelf that also reduce vessel attractiveness and the risk of incidental mortality, would be of high overall conservation benefit.

## Introduction

Fishing vessels represent important sources of food for many seabird populations, through the provision of offal and discards following on-board processing, baits removed during long-line setting, and fish lost or taken from the net during trawling [1]–[3]. Such supplementary food may not always have positive effects on seabirds, as proposed by the “junk-food” hypothesis [4]. Fisheries may also cause significant seabird mortality (through accidental death in fishing gear) and, potentially, compete with seabirds for prey [5]–[7]. Many seabirds, including 21 out of 24 extant albatross species, have an unfavourable conservation status, with the main identified threat being mortality through fisheries bycatch [8]. Hence, understanding the modes by which birds interact with fisheries is highly relevant, for conservation, and for a better understanding of seabird spatial ecology and demography.

Considerable efforts have been exerted to document the degree of overlap between susceptible seabirds and fishing fleets, and to quantify the amount of food provided by fisheries discards [9]–[12]. Most studies to date have addressed this issue at relatively coarse spatial and temporal scales, or had access to tracking data from few birds [11],[13]–[17]. Despite all the interest, very little is known about the ways in which individuals interact with fishing boats, whether particular birds specialise in following ships, the proportion of the population that does so, and how much time is spent in association with vessels on typical foraging trips.

Black-browed albatrosses (*Thalassarche melanophris*) are considered to be *Endangered* on a global scale [8]. Their main breeding stronghold is in the Falkland Islands, and birds from these colonies forage mostly over the Patagonian shelf [18], [19] where they overlap with several fishing fleets. During early chick-rearing, the most active fishery in their foraging range is the finfish trawler fishery. Albatrosses interacting with this fishery are known to suffer from considerable mortality due to accidental collisions with warp cables [20], [21].

The marine waters under the jurisdiction of the Falklands provide a particularly favourable context in which to study the details of the interaction between albatrosses and fisheries, because there is virtually no artisanal fishing and all fisheries activities are monitored through a Vessel Monitoring System (VMS). We took this opportunity to, for the first time, precisely determine the level of spatial and temporal interactions of albatrosses and fishing vessels. Here, we demonstrate that by tracking birds and vessels simultaneously using GPS technology, we can provide answers to the following questions: (1) Do birds from different colonies differ in the propensity to follow vessels? (2) How often and for how long do individual birds associate with fishing vessels? (3) Do some individual albatrosses consistently associate with fishing vessels, whereas others avoid or ignore them?

## Methods

### Ethics Statement

The deployment of the GPS and MK7 loggers (see details below) did not took more than 10 minutes and on no occasion did birds leave the nest as a result of handling. Also, not a single nest failed due to desertion during this study or in the week after it was completed. The work was approved by the Falkland Islands Environmental Committee. All deployments were carried out under permissions issued by the Falkland Islands Government, Environmental Planning Department (Research Licenses number R09/2008 and R12/2009).

### Falkland Islands trawl fisheries

The fishing vessels operating in the Falkland Islands are freezer/factory bottom trawlers targeting a variety of finfish species, including Patagonian rock cod (*Patagonotothen ramsayi*), hoki (*Macruronus magellanicus*), hakes (*Merluccius hubbsi* and *M. autralis*), southern blue whiting (*Micromesistius australis*), red cod (*Salilota australis*) and kingclip (*Genypterus blacodes*). Most vessels are Falkland Islands or Spanish flagged. Fewer Korean vessels operating in the area target skates (Rajidae). The important squid fishery using jiggers only operates in the austral autumn/winter, outside the albatross nesting season. In the summer, finfish vessels typically trawl an average of 14 hours per day. Individual trawl duration can vary between 1–5 hours, depending on how quickly the net is filled up. Finfish are processed into various products, including headed and gutted trunks, fillets and skate wings. As a result offal and heads are discarded. Vessels also discard unwanted by-catch and this includes undersized fish. The fishery has been targeting rock cod (*P. ramsayi*) for the last three years and in 2010 the catch reached 76,723 metric tonnes. Vessels generally retain individuals with total length in excess of 25 cm and as a result c. 20% of this catch is discarded. The Falkland Islands Government Fisheries Department (FIFD) is currently examining ways to reduce this volume of discard by reviewing codend mesh sizes within the fishery. Substantial volumes of discarded by-catch, heads and offal attract large numbers of seabirds which feed on the discard as it moves around to the stern of the vessel. As the vessel pitches in moderate to heavy seas, the 50 mm diameter steel warps cut through the water with sufficient speed to trap or entangle birds foraging at the stern of the vessel, forcing them under water and causing death by drowning or serious injuries leading to delayed fatality. As a consequence of this, in 2004, the FIFD made it a licence condition that all trawlers operating in Falkland Islands waters and all Falkland Islands registered vessels operating outside the Falkland Islands use a tori line over each warp when trawling is in operation in order to mitigate seabird mortality.

### Albatross tracking data

This study took place on New Island (2008/09 and 2009/10) and on Steeple Jason (2009/10), West Falkland Islands. New Island (51°43′S, 61°18′W) holds ca. 12,000 breeding black-browed albatross pairs, and Steeple Jason (51°01′S, 61°13′W) holds ca. 180,000 pairs, representing the largest colony in the world for this species.

Adult breeding birds were tracked during early chick-rearing in December-January using GPS loggers (Earth & OCEAN Technologies), attached to the back feathers with Tesa tape. Birds were also fitted with a British Antarctic Survey Mk7 logger (3.5 g) on a plastic leg band, to record the timings of all changes of immersion state (from wet to dry, and *vice versa*, with 3 s resolution) allowing the reconstruction of detailed activity patterns.

GPS loggers accurately recorded the position of the study birds every 7 or 14 minutes (depending on the size of the battery attached to the device, resulting in a mass of 25 g or 30 g, respectively). Information on each of the fishing vessels operating inside Falkland Island waters was obtained through VMS records that provided the exact position of each vessel every 3 h (2008/09) or every 1 h (2009/10). Additionally, vessel positions were obtained every 30 min on 27 December 2009. Positions of fishing vessels and albatrosses were linearly interpolated to 3 s, to match the temporal resolution of activity records (see Video S1).

All birds that approached vessels did so in rapid flight and the beginning of an interaction was defined as the moment when the bird first landed within 3.5 km of the ship, or when the estimated distance between bird and ship was <0.5 km. An interaction ended when the bird took off and flew away from the ship, or when the ship travelled a distance of 5 km from a bird that remained on the water. Encounters when birds never landed close to the vessel did not meet these interaction criteria, and were not considered further (such cases were extremely rare). The above criteria defined *single* interactions. Sometimes birds remained in the general vicinity of a ship (or group of ships) after the end of an interaction, only to approach the same vessel(s) again, minutes or (up to 8) hours later; hereafter, these are referred to as *serial* interactions.

Energetic cost of take-off is high in albatrosses, and it can be assumed that most landings are attempts at prey capture, unless prior to prolonged periods (several hours) spent resting on the water at night [22], [23]. Landing events on the water therefore provide a good indication of the distribution of foraging effort. Landings at <1500 m from the breeding colony were excluded from all summarizing statistics, as albatrosses often bathe, or preen, but rarely forage, in the immediate vicinity of the nesting site.

The spatial distribution of landings on the water was described using kernel home range utilization distribution (smoothing factor selected using the “*ad-hoc* method”, [24]) following a Universal Transverse Mercator projection of the albatross positions estimated each 3 s. Only the initial position of each wet bout was included in this analysis. Kernels density maps of fishing effort of the Falklands fleet were based on hourly positions of vessels moving at <6 knots. This speed filter excludes the majority of vessels in rapid transit between hauling stations [25]. We calculated the kernel overlap between seabirds and fisheries using the utilization distribution overlap index [24].
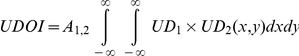
where A_1,2_ is the area of overlap between the distribution of seabirds and fisheries, UD_1_ and UD_2_ are the corresponding utilization distributions, *x*, *y*, *dx* and *dy* are the longitude, latitude, delta longitude and delta latitude, respectively.

To compare the search strategies adopted in trips with and without interactions with vessels, we calculated a straightness index, computed as the ratio between twice the straight line distance from the colony to the farthest point of the trajectory and the total trip length [26]. To improve the comparability of the straightness index between trips with and without interactions, we only included trips in which the maximum distance reached from the colony was equal or greater than the distance from the colony to the nearest fishing vessels.

For the purposes of this study, trips with incomplete information (due to battery depletion) were excluded from analysis. Means are presented with standard deviations. Nautical twilight times were calculated from http://www.usno.navy.mil/USNO/astronomical-applications/data-services/rs-one-year-world.

## Results

**Figure 1:**
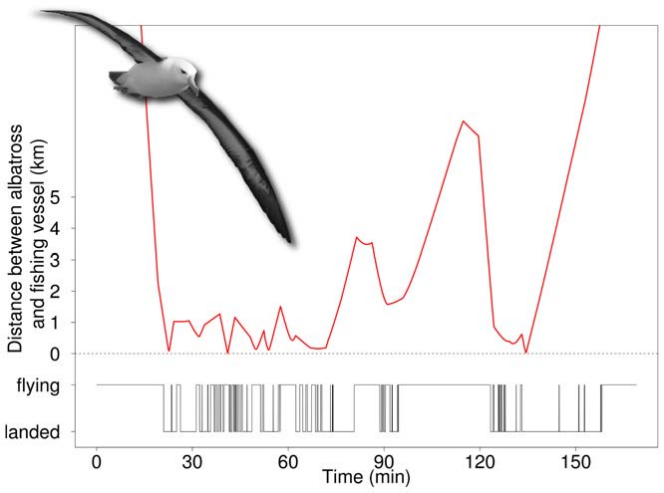
Activity patterns (time spent in flight *vs*. on the sea surface) of one tracked albatross in relation to distance from the fishing vessel.

We tracked a total of 173 trips from 99 individual albatrosses (Table 1). The high spatial and temporal resolution of tracking data from birds and vessels allowed proximity to be measured accurately (see Methods S1), and, in combination with the immersion (activity) data, the detailed analysis and interpretation of interactions between birds and vessels (Fig. 1). This was further facilitated by the production of animations representing the simultaneous positions of birds and vessels (see Video S1).

**Table 1:** Characteristics of foraging trips during early chick-rearing of black-browed albatrosses tracked from New and Steeple Jason islands in 2008 and 2009.

	2008	2009	2009	Overall
	New Island	New Island	Steeple Jason	
Number of individuals tracked	39	35	25	99
Total complete trips	72	65	33	170
Complete trips inside Falkland waters	39	17	21	77
Trip length (km)	780±57	859±422	547±404	
	(72)	(65)	(33)	
Trip length (km) *	370±226	313±103	306±137	340±184
	(39)	(17)	(21)	(77)
Trip duration (hours)	51.8±31.0	56.1±26.0	53.0±26.1	53.7±28.2
	(72)	(65)	(33)	(170)
Trip duration (hours) *	37.1±21.3	38.9±10.9	41.9±18.7	38.8±18.7
	(39)	(17)	(21)	(77)
Number of landings on the sea per trip	133±79	136±69	120±63	132±72
	(72)	(65)	(33)	(170)
Number of landings on the sea per trip *	128±71	124±72	112±58	122±68
	(39)	(17)	(21)	(77)
Percentage of trip time spent inside Falkland waters	74±31	53±32	87±21	
	(72)	(65)	(33)	
Complete trips inside FICZ with interactions (all)	4/39	3/17	8/21	
Complete trips inside FICZ with interaction (only one – first- per ind))	2/25	2/12	4/13	
Percentage of time spent interacting with ships	0.3±1.5	0.9±4.0	2.3±9.9	
	(72)	(65)	(33)	
Percentage of time spent interacting with ships *	4.4±1.9	3.3±7.5	2.9±5.7	
	(39)	(17)	(21)	
Percentage of landing events in the vicinity of ships	1.0±4.2	2.3±9.9	8.8±18.2	
	(72)	(65)	(33)	
Percentage of landing events in the vicinity of ships *	1.3±5.5	7.6±18.4	12.4±21.8	
	(39)	(17)	(21)	

An overall mean is presented when there are no statistically significant differences between years or colonies. Values represent mean ± SD (sample sizes in parenthesis). Characteristics marked with * were based only on trips in which the tracked birds remained entirely within Falkland Islands waters.

During 78 trips (45% of the total), involving 50 individuals, tracked birds never exited the marine areas under Falkland's jurisdiction (designated as Falkland Islands Interim Conservation Zone (FICZ) and Falkland Islands Outer Conservation Zone (FOCZ)). Interactions with tracked fishing vessels occurred in only 19% of these 78 trips, and 16% of a sub-sample of 50 statistically-independent trips, involving the first or only trip from each individual (see also Table 1). Relatively few trips inside Falkland waters involved an association with a fishing vessel, but even when these were recorded (in 15 trips in total), the proportion of time spent interacting was small, representing an overall value of 9.0±7.9% (range: 0.1–24.0%) of the time away from the colony, and 29.4±22.9% of landings (range: 0.4–79.0%), which we considered an indicator of foraging activity (see above). Considering all trips in Falklands waters, only 5.7±15.3% of all landings were made in the immediate vicinity of ships (see also Table 1).

The mean duration of all *single* interactions was 1.1±1.1 hours (N = 56), and ranged from 3 minutes to just under 7 hours. A few individuals engaged in *serial* interactions, temporarily settling in an area with several fishing vessels, amongst which they commuted, possibly obtaining food and pausing between foraging bouts. For example, on 26–27 December 2009, bird 1438019, from Steeple Jason, remained in an area of ca. 500 km^2^ for 18.5 hours, interacting on six occasions with 3 vessels. Considering *serial* interactions as separate sampling units, the total number of all interactions (*single* and *serial*) is reduced to 30, with a mean duration 5.4±7.1 hours, and a median of 2.1 hours (range 9 minutes to 25 hours).

**Figure 2:**
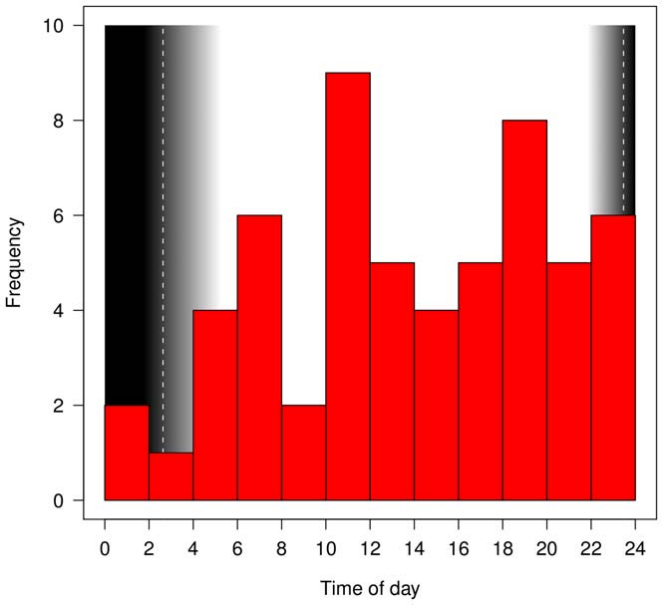
Distribution of times (local time, GMT-3h) of start of interactions between albatrosses and fishing vessels. Vertical dashed lines indicate the nautical dusk and dawn calculated for 19 December. Black indicates night and grey indicates twilight.

In the 15 trips confined to Falklands waters that involved a bird-vessel interaction, the landing rate was much higher during the periods of interaction than at other times (9.1±3.9 *vs*, 2.2±1.4 landings.hour^−1^; P<0.001). During interactions, birds spent ca. 75% of the time on the water. Interactions between birds and ships were more frequent during daylight than darkness (Fig. 2).

**Figure 3:**
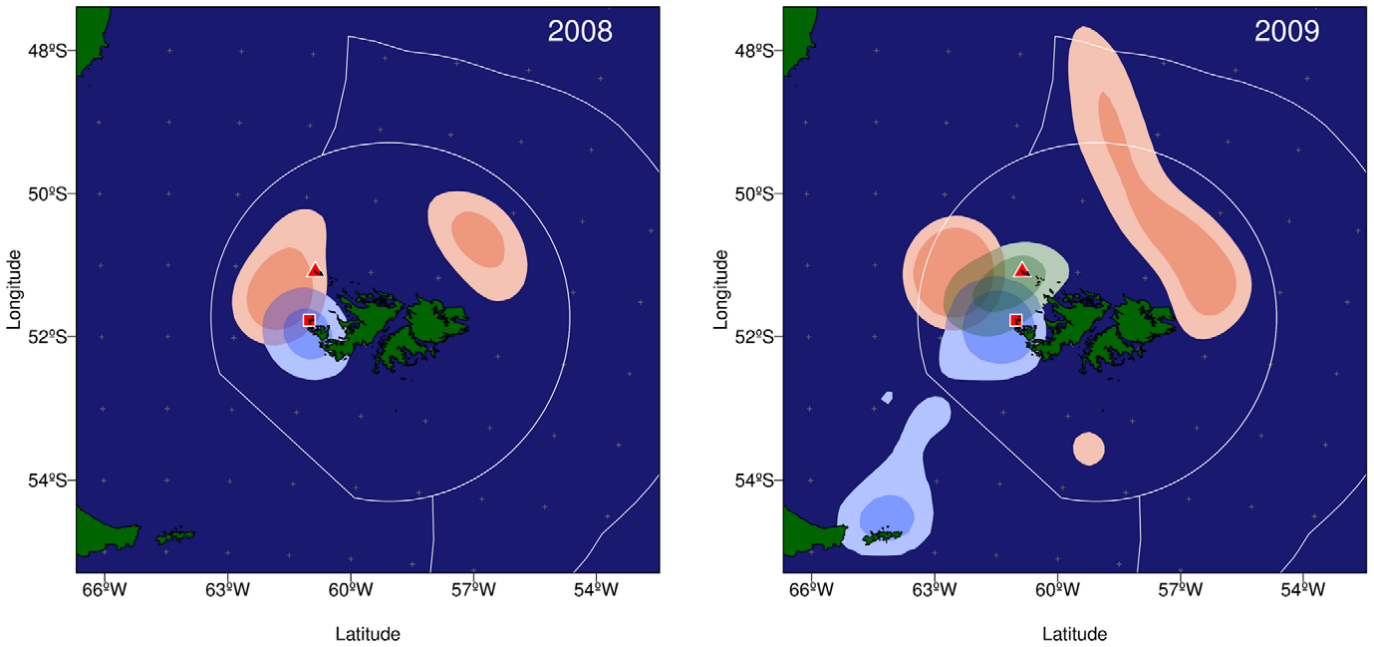
Distribution of fishing effort (pink), and foraging activity (inferred from landings on the water) of albatrosses from New Island (blue) and Steeple Jason (green) in 2008 and 2009. Dark and light shades represent 25% and 50% kernel utilization, respectively. Red triangle indicates the location of Steeple Jason and the red square indicates New Island. White lines indicate the limits of the inner and outer Falklands Interim Conservation Zone.

In 2009/10, the proportion of trips during which the birds interacted with the Falklands fishing fleet differed between the study colonies: considering only one (the first) trip per individual, the value was 2 of 35 = 0.06 for New Island and 7 of 25 = 0.28 for Steeple Jason (Fisher's Exact Test: P = 0.027). This was unrelated to the distance from the study colonies to the vessel fishing grounds. The distance separating the study colony on New Island from the centre of the distribution of the fishing effort west of the Falklands (defined by the centroid of the 25% probability contour provided by the kernel) was 61 km in the first year and 125 km in the second. The corresponding values for Steeple Jason were 59 and 110 km. The differences in overlap between the fishing fleet and tracked birds from each colony in 2009 also indicates a higher degree of interaction by birds from Steeple Jason, compared with New island (overlap = 0.408 and 0.216 respectively; Fig. 3).

Of the 67 individual albatrosses that were tracked twice, only 3 birds (<5% of the total), all from Steeple Jason, visited the Falklands fishing fleet on both trips. In each case, the birds returned to one or more specific vessels with which they had interacted in a previous trip. The level of association with vessels in the 6 trips made by those 3 individuals was no different to that in the other 9 trips with interactions made by other birds: proportion of the time engaged in interactions, 8.3±8.5% *vs.* 9.4±8.0%, and percentage of landings near fishing vessels 30.0±20.8% *vs.* 28.5±27.7%. Of individual trips where interactions with ships were recorded, 19 involved interactions with only 1 vessel, 4 with 2, and 4 with 3 vessels.

Trips in which the fishing fleet was visited were no different in duration, distance covered, straightness index or number of landings from trips where no interactions with vessels were recorded (Table 2).

**Table 2:** Comparison of characteristics of foraging trips of black-browed albatrosses inside the Falkland islands' waters in which the birds did, or did not, interact with the fishing fleet.

	Trips without	Trips with	Comparison
	vessel interaction	vessel interaction	
Trip duration (hours)	38.6±19.5	39.5±15.0	F_1,76_ = 0.43
	(63)	(15)	P = 0.52
Trip length (km)	322±193	415±111	F_1,76_ = 3.14
	(63)	(15)	P = 0.08
Number of landings	125±68	113±65	F_1,76_ = 0.03
	(63)	(15)	P = 0.87
Straightness index	0.500±0.178	0.596±0.202	F_1,53_ = 2.96
	(40)	(15)	P = 0.87

Values represent mean ± SD (sample sizes in parenthesis).

## Discussion

This is the first study published to date to use high resolution tracking data from both birds and vessels to provide a comprehensive picture of the nature and extent of fisheries interactions for an archetypal scavenging seabird, the endangered black-browed albatross. As never before, the amount of time spent in association with fishing vessels at the individual and population level is precisely quantified. In addition to greatly improving knowledge of the level of reliance of seabirds on fisheries waste, given that incidental mortality risk will at least to some extent be a function of the amount of time spent in the vicinity of fishing vessels [27], the type of data presented here is integral to the development of effective models of spatial and temporal variation in fisheries-related threats [28].

**Figure 4:**
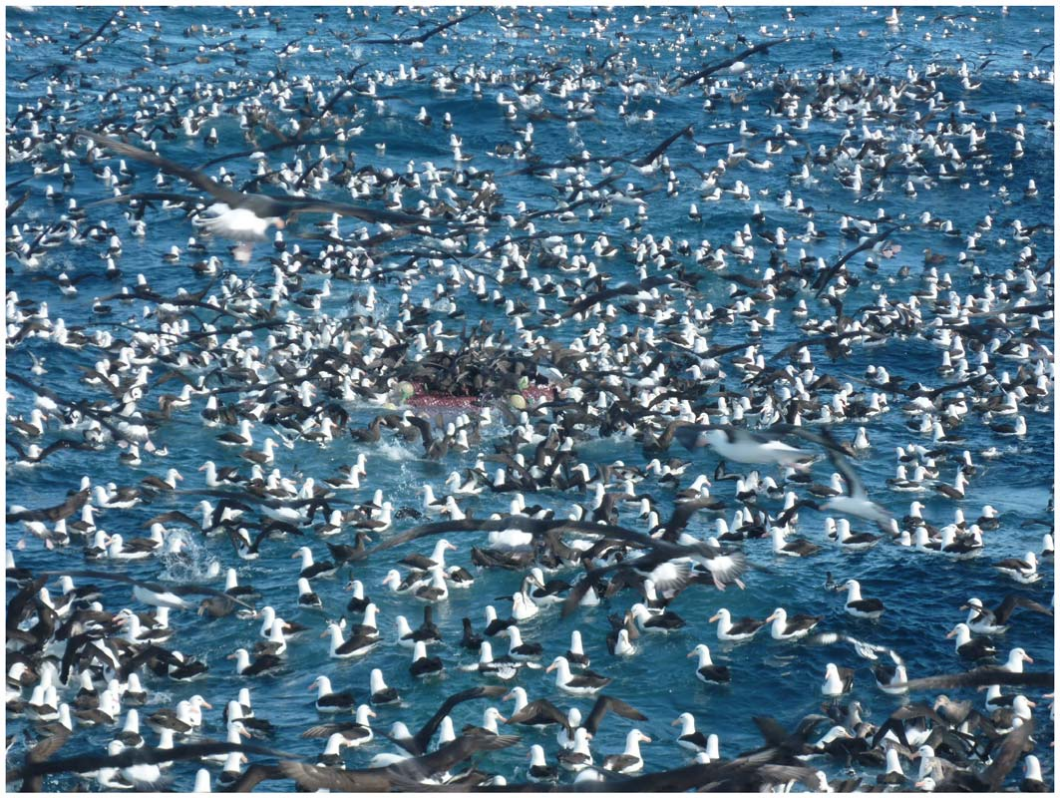
Albatrosses scavenging behind fishing vessel in Falkland Island waters.

By producing animations that reconstructed the activities and spatial interactions of birds and ships, we were able to readily visualise and interpret events taking place far offshore (see Video S1). This level of insight cannot be achieved using vessel-based observers, given their limited capacity to follow closely the behaviour of single birds amidst the multitude present during fishing operations (Figure 4), and given the total lack of knowledge of movements and activity of those individuals when not following vessels. Furthermore, onboard observations cannot provide information about the colony of origin of birds attending fishing vessels. Meaningful animations were produced despite intervals of 0.5–3.0 h between the vessel positions available from the VMS, as the errors in interpolated ship locations remained relatively low (see Methods S1). The inclusion of data on bird activity (time spent in flight *vs*. on the water) provided an important complementary element in the interpretation of the spatial data provided by the GPS loggers, and proved invaluable for identifying genuine bird-vessel interactions.

A previous study by Otley et al. [16] of wandering albatrosses *Diomedea exulans* in the South Atlantic combined satellite-tracking data from birds with vessel-based observations. Data available from two complete foraging trips indicated that individual albatrosses spent several days (around half the time spent at sea) in association with the vessels. Our much finer resolution data suggest that at least for black-browed albatrosses around the Falklands, the modes by which birds associate with vessels may be considerably different. Most interactions were relatively short, usually a few minutes to a few hours, and only a small percentage of the total time spent at sea and of foraging effort (inferred from landings) was made in actual association with fishing vessels. Furthermore, trips in which interactions with vessels occurred were no different from all others in terms of distance covered, duration, path straightness or foraging effort. Despite the fact that 3 individuals visited vessels in consecutive foraging trips, we did not gather conclusive evidence that attending fishing vessels represents a distinct foraging strategy, in terms of the characteristics of the travel path or activity while at sea. This, in addition to the limited proportion of trips with interactions, and the relatively short time periods spent in association with fishing vessels, supports the idea that discards and offal are relatively unimportant in energetic terms for the study population during early chick rearing.

How can these observations and deductions be reconciled with the hundreds to thousands of albatrosses often seen attending individual ships on the Patagonian Shelf (Figure 4), as well as with inferences from studies in the same broad region [3], [29] that black-browed albatrosses are likely to consume large quantities of fisheries discards when these are available? First, it must be kept in mind that albatrosses from different colonies may show contrasting levels of association with fisheries (see below). Secondly, the Falkland Islands black-browed albatross population (including breeders, adult non-breeders and immatures), is likely to exceed 1 million individuals (calculated from data in ACAP [19]); hence, even if individual albatrosses follow fishing vessels only infrequently, it is still possible that the aggregations are impressive when they do so.

This preliminary study covers only a limited part of the annual cycle of black-browed albatrosses. However, early-chick rearing is the most sensitive phase of the breeding cycle of *Thalassarche* albatrosses, when daily nest failure rate is greatest ([30] and own. unpubl. data). The weak association with fisheries at this stage therefore hints at a low level of dependency on fisheries waste by black-browed albatrosses from the Falklands' population, potentially throughout the breeding season. Although this may seem surprising (see above), it accords with the low proportion (just 4.4%) of the population energy requirements that finfish fisheries discards were considered to form in the early 1990s, on the basis of discard levels and bioenergetic calculations [9]. An important conservation consideration is that any measures that reduce the availability of discards for birds on the Patagonian shelf will be beneficial. In fact, given their apparent low dependency on fishery waste, the impact on food supply is likely to be minimal, but the attractiveness of vessels, and hence the likelihood of incidental mortality through interaction with fishing gear, should be reduced substantially [19], [31]. This is the same conclusion reached by Petersen et al. [32] for the Benguela upwelling system where, similarly, black-browed albatrosses spend considerably more time feeding on natural prey than in association with fisheries. Without such measures, the Falklands black-browed albatrosses will remain highly susceptible to incidental mortality, as this species is known to be caught in considerable numbers in longline and trawler fisheries on the Patagonian Shelf [7], [19], [20], [33]. This suggests that either levels of interaction with these fisheries increase during the nonbreeding period beyond those observed in our study, or if not, that even limited periods spent behind vessels by a small proportion of the population represents an important mortality risk. The latter might well involve the same individuals that exhibited a consistent tendency to associate with fishing vessels in this study.

Another important finding of the present study relates to the differences in behaviour of albatrosses originating from the two colonies, New Island and Steeple Jason. The proportional contribution of birds from these adjacent islands (separated by 75 km) to the pool attending a particular fishing fleet could not have been anticipated simply from relative distance to the main fishing areas; the centroid of the nearest area of fishing effort (based on the kernel analysis) was almost equidistant to the two colonies, and yet the birds from Steeple Jason were 4.5 times more likely to attend those fishing vessels. As Steeple Jason has ca.15 times as many nesting albatrosses as New Island, any albatross seen attending those vessels was 68 times as likely to be from Steeple Jason as from New Island. Although the spatial segregation of flying seabirds from neighbouring colonies has been documented before [18], [34], existing studies generally focused on colonies that were more widely separated (relative to foraging distance), and did not analyse the phenomenon in relation to fisheries. Our data do not enable any supported explanation for the difference in vessel attendance between New Islands and Steeple Jason albatrosses. A preliminary (unpublished) analysis of movements and habitat selection of black browed albatrosses from these colonies suggest that most tracked birds avoided leaving the colony by flying into the wind, which was predominantly from the NW during this study (see Fig. 3). This might account for the paucity of birds from New Island around the fishing fleet, but with the available data any explanation remains a matter for speculation. Further research is needed to clarify this issue.

In conclusion, the simultaneous, high resolution, tracking of seabirds and fishing vessels, such as in the present study, offers huge potential for new insights into the ecology of threatened species, their relationships with human activities and improved application of ecosystem-based fisheries management practices at wide spatial and temporal scales.

## Supporting Information

Methods S1 **Errors in distances between albatrosses and ships.** Details of the computation of errors involved in the estimation of distances between albatrosses and ships;
(DOC)

Video S1 **Animation of albatross interacting with fishing vessel (best viewed with a web browser).** The animation was produced from Vessel Monitoring System (positions every 1 hours) and bird GPS (positions obtained every 14 minutes) data and from inferred positional data every 3 seconds obtained through linear interpolation between known fixes. The albatross track is depicted in red when the activity logger data indicated the bird to be sitting on the sea surface. Note the clock (fast) running on the upper corner of the screen. Note that at the beginning of the animation, a ship sails past the focal albatross, which is sitting on the sea surface, without eliciting any response. Latter, the albatross approaches and follows a ship, landing in its vicinity several times. Animations such as the present one were produced for each occasion a study albatross came within an estimated 10 km from any fishing vessel operating in marine waters under Falklands jurisdiction.
(GIF)
