# Supplementary material for: Albatrosses Following Fishing Vessels: How Badly Hooked Are They on an Easy Meal?
Source: PLoS One. 2011 Mar 2;6(3):e17467. doi: 10.1371/journal.pone.0017467 (PMC3047564; doi:10.1371/journal.pone.0017467)
Supplement: Methods S1 — Errors in distances between albatrosses and ships. Details of the computation of errors involved in the estimation of distances between albatrosses and ships; (DOC) [file pone.0017467.s001.doc]

**Methods S1: Electronic supplementary material.**

**Errors in distances between albatrosses and ships.** Details of the computation of errors involved in the estimation of distances between albatrosses and ships

The errors of the positions directly provided by the GPS loggers or the VMS are negligible. As explained in the Methods, albatross positions were obtained every 7 minutes or every 14 minutes, depending on the GPS battery, while vessel positions were obtained every 30, 60 or 180 minutes. Positions every 3 seconds were obtained through linear interpolation. We estimated the magnitude of the likely errors for these every 3 second interpolated positions through the following procedure.

For albatrosses with positions every 7 minutes, we discarded every second GPS position, calculated it through linear interpolation between the two nearest positions, and computed the distance between the estimated position and the actual coordinates obtained from the loggers. This gives the error (εbird_7_min) of the most temporarily distant points from known positions. At the relevant scale, the magnitude of the position error is likely to be approximately linearly related to the time distance between the relevant point and the nearest known position. Hence, the mean error for any i position of a bird with 14-min record intervals is halfway between the maximum and the minimum error (which is assumed to be zero). So, to the pool of N εbird_7_min estimates, from which we randomly sampled values (see below), we added N+1 zero values, to obtain a ε_bird_ with a mean value representative of all bird positions. A similar procedure was adopted to calculate ship estimated position errors.

To calculate the mean error of the distance estimates between ships and birds, we created 1000 pairs of vectors (one ship vector + one bird vector) with the same origin and each with a randomly assigned direction. The size of the bird vector was obtained randomly sampling from the ε_bird_ distribution, and similarly for ship errors. The distribution of the lengths of the vectors resulting from the addition of the two vectors in each pair gives a rough estimate of the distribution of the errors in distance estimates. The mean error (± SD) of the distances for ship and albatross positions at 1h and 14 min intervals, respectively, was 1.15 ± 1.44 km; with vessel positions obtained every 3h the error was 3.42 ± 3.29km.

Note that half of the birds tracked in this study were sampled at 7-minute intervals and all ships in the second study year were sampled at 1-hour intervals or less. Hence, the small errors calculated above apply to worst case situations only. In most cases, the errors must have been considerably smaller than those. Furthermore, almost all interactions lasted for considerably longer than the longest sampling interval for albatrosses, and many lasted longer than the sampling interval for ships, which implies that at least one bird position per interaction was virtually error-free and the same applies to a majority of ship positions. Considering the above calculations and considerations and the quality of the reconstructions of albatross and vessel tracks (see animation in electronic supplementary material S2), it seems unlikely that any study albatross *versus* ship interaction might have been overlooked by us during this study.
