# Supplementary figures and images for: Albatrosses Following Fishing Vessels: How Badly Hooked Are They on an Easy Meal?
Source: PLoS One. 2011 Mar 2;6(3):e17467. doi: 10.1371/journal.pone.0017467 (PMC3047564; doi:10.1371/journal.pone.0017467)

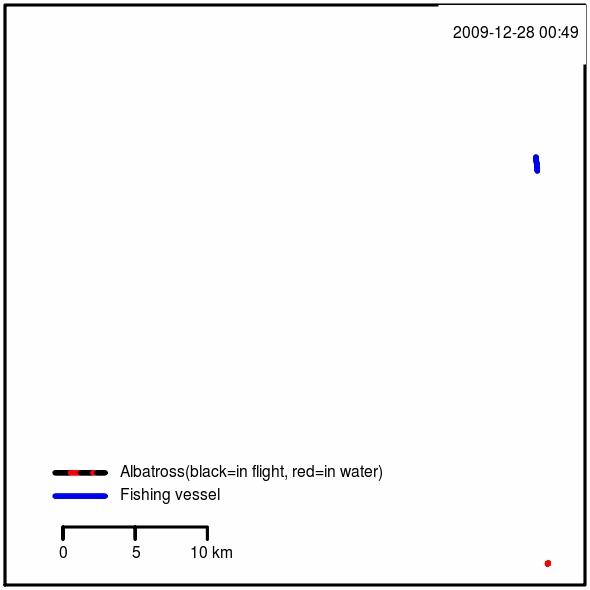

Supplement: Video S1 — Animation of albatross interacting with fishing vessel (best viewed with a web browser). The animation was produced from Vessel Monitoring System (positions every 1 hours) and bird GPS (positions obtained every 14 minutes) data and from inferred positional data every 3 seconds obtained through linear interpolation between known fixes. The albatross track is depicted in red when the activity logger data indicated the bird to be sitting on the sea surface. Note the clock (fast) running on the upper corner of the screen. Note that at the beginning of the animation, a ship sails past the focal albatross, which is sitting on the sea surface, without eliciting any response. Latter, the albatross approaches and follows a ship, landing in its vicinity several times. Animations such as the present one were produced for each occasion a study albatross came within an estimated 10 km from any fishing vessel operating in marine waters under Falklands jurisdiction. (GIF) [file pone.0017467.s002.gif]
